# Bibliometric analysis of RNA-binding proteins in osteosarcoma: unraveling research trends and hotspots

**DOI:** 10.3389/fimmu.2025.1577261

**Published:** 2025-09-12

**Authors:** Zhiqian Gu, Songou Zhang, Xudong Hu, Nanjian Xu, Yang Wang, Jian Ruan, Yufeng Qian, Weihu Ma, Hong Chen

**Affiliations:** ^1^ Health Science Center, Ningbo University, Ningbo, Zhejiang, China; ^2^ Department of Orthopaedics, Ningbo No.6 Hospital, Ningbo, Zhejiang, China; ^3^ Department of Neurosurgery, Shaoxing People’s Hospital, Shaoxing, Zhejiang, China

**Keywords:** osteosarcoma, RNA-binding protein, bibliometric, non-coding RNA, bone tumor

## Abstract

**Background:**

RNA-binding proteins (RBPs), a class of molecules that play a crucial role in regulating gene expression, have attracted considerable attention in cancer biology research. RBPs influence osteosarcoma progression by modulating RNA metabolism and participating in cellular proliferation, differentiation, apoptosis, and interactions within the tumor microenvironment. Understanding the current status and future trends of RBPs is crucial for the advancement of osteosarcoma research.

**Methods:**

Relevant literature was sourced from the Web of Science, PubMed, and Scopus databases covering the period from January 1, 1994, to December 31, 2024. Using professional analytical tools such as R bibliometrix, VOSviewer, CiteSpace, and SCImago, we conducted a multidimensional visual analysis of publication trends, contributions from countries and institutions, influential authors, significant publications, and keyword distribution.

**Results:**

Research on RBPs in osteosarcoma began in 1994, with a notable increase in published studies since 2016. The leading countries for research output were China and the United States, primarily from three major U.S. institutions: the University of Illinois, Harvard University, and UT MD Anderson Cancer Center. Significant contributors to this field included Kannanganattu V. Prasanth, Jean-Yves Masson, Yang Wang. The most cited article was a review titled *The potential role of RNA N6-methyladenosine in Cancer progression* by Professor Shaoqing Ju from China (2020). Prominent journals within this domain included *Cancer Research* (USA), *Oncogene* (England), *Cancer Cell International* (England), and the *Journal of Bone and Mineral Research* (USA).

**Conclusion:**

This study highlights the critical role of RBPs in osteosarcoma. We conducted a systematic literature review using bibliometric methods to outline the research landscape, identify hotspots and emerging trends, and provide valuable references for future studies. Future research should focus on enhancing international collaboration, exploring molecular mechanisms, and connecting these insights to clinical applications—especially in targeted drug development—to improve treatment outcomes for osteosarcoma patients.

## 1 Introduction

Osteosarcoma is the most common primary malignant bone tumor, with a high incidence among children and adolescents, significantly impacting patients’ physical health and quality of life (1–4). The incidence of osteosarcoma exhibits a bimodal distribution. The first peak occurs in adolescents aged 10 to 19 years, with an incidence rate of 4.9 to 10.8 cases per million annually (5). The second peak is observed in individuals aged 60 to 79 years, with a lower incidence of 1.7 to 4.9 cases per million individuals per year (5). Although there has been some progress in treatment, including optimized surgical techniques, the improvement of chemotherapy drugs, and the refinement of radiotherapy regimens, the 5-year survival rate for patients with metastatic or recurrent osteosarcoma remains low. Reports indicate that the overall 5-year survival rate ranges from 49% to 58% (6, 7). Additionally, the incidence of osteosarcoma has increased significantly in recent years (8). This trend highlights the urgent need for innovative research to explore new treatment strategies and improve patients’ prognosis.

In cancer biology, RNA-binding proteins (RBPs) have emerged as a crucial research focus in recent years (9–12). These proteins play a critical role in post-transcriptional regulation (13), precisely controlling various aspects of RNA metabolism, including splicing, polyadenylation, transport, localization, stability, and translation (14, 15). By recognizing and binding to specific RNA sequences or complex RNA structures. They can both enhance and inhibit the expression level of target genes. This regulatory effect has a profound impact on a series of fundamental biological processes such as cell proliferation, differentiation, apoptosis, and migration (16–18).

In osteosarcoma research, RBPs are closely related to the occurrence and development of tumors (19). Studies have shown that abnormal expression or dysfunction of RBPs is significantly associated with aggressive behaviors in osteosarcoma cells, including increased proliferation, invasiveness, metastatic potential, and resistance to chemotherapeutic drugs (20, 21). For example, MSI1 promotes osteosarcoma cell cycle progression by activating key regulatory factors such as p21 and p27, thereby accelerating cell division and proliferation (22). Additionally, HuR can promote epithelial-mesenchymal transition (EMT) in osteosarcoma cells by regulating the expression of genes related to cell adhesion, including E-cadherin and N-cadherin, thereby facilitating tumor cell migration, invasion, and metastasis to surrounding tissues and distant organs (23).

RBPs play an indispensable role in the complex interaction network of the tumor microenvironment (24–27). This microenvironment is composed of multiple components, such as tumor cells, immune cells, fibroblasts, vascular endothelial cells, and the extracellular matrix (28). RBPs regulate osteosarcoma cells to secrete a variety of cytokines, chemokines, and growth factors, which attract immune cells to the tumor site, modulate their activity and function, and contribute to immune evasion (29, 30). Additionally, RBPs stimulate the proliferation and migration of vascular endothelial cells, promoting tumor angiogenesis, providing tumor cells with sufficient nutrients and oxygen supply, and further supporting tumor growth and metastasis (31, 32). RBPs play a pivotal role in epigenetic regulation, particularly within the burgeoning field of epitranscriptomics, which is concerned with chemical modifications of RNA. Among these modifications, N6-methyladenosine (m6A) stands out as the most prevalent and well-characterized (33–35). RBPs dynamically modulate the addition, recognition, and removal of m6A modifications, thereby influencing mRNA stability, translation efficiency, and splicing (36–39). Current research indicates that RBPs play a significant role in the development of osteosarcoma by modulating m6A methylation of various mRNAs and non-coding RNAs. As a m6A eraser and RBP, ALKBH5 increases SOCS3 mRNA expression by reducing its m6A modification, inhibiting osteosarcoma cell proliferation and promoting apoptosis (40). WTAP, as a m6A writer, enhances circ_0032463 expression through m6A methylation, which fosters growth and metastasis in osteosarcoma cells (41). Additionally, NAT10 boosts glycolysis and promotes osteosarcoma cell growth via m6A modification mediated by the reader protein YTHDC1 (42).

Given the critical role of RBPs in osteosarcoma research, it is particularly important to fully and deeply understand the current status and development trend of research in this field. Bibliometric analysis, a big-data-driven research evaluation method, enables the systematic exploration of the historical evolution, research hotspots, future directions, and cooperation in RBP-related osteosarcoma studies. Through systematic analysis of key information such as publication timelines, authors, institutions, countries, keywords, and citation frequency of the literature, this approach helps identify core research areas, capture emerging trends, and highlight influential authors, institutions, and countries. Additionally, it offers insights into collaborative networks and the temporal evolution of research topics, thereby providing a clear perspective on the development of the field (43–46). In the present study, we aimed to systematically organize existing literature on RBPs in osteosarcoma, accurately identify research hotspots and emerging trends, and provide a comprehensive, in-depth, and forward-looking reference for future research, ultimately advancing developments in this field.

## 2 Methods

### 2.1 Information sources and eligibility criteria

This bibliometric analysis was conducted in accordance with the PRISMA 2020 guidelines for data extraction and analysis (47). In this study, we selected three databases—Web of Science, PubMed, and Scopus—for literature retrieval and data collection. The Web of Science is widely recognized as the oldest and most authoritative database for research publications and citations, encompassing extensive coverage of approximately 34,000 premier journals globally (48). Web of Science offers a Core Collection with high-quality citation data and metadata crucial for bibliometric analysis. These datasets integrate seamlessly with notable bibliometrics software, enabling researchers to analyze academic trends, explore collaboration networks, and identify emerging hotspots across various fields. PubMed is the most widely used search engine for biomedical literature and is the preferred database for many researchers in health and life sciences (49). Scopus, launched by Elsevier in 2004, is a comprehensive abstract and citation database (50). Like the Web of Science, it hosts numerous high-quality documents across various scientific disciplines (51).

Keyword searches were conducted in Web of Science, PubMed, and Scopus to identify relevant literature within the timeframe from January 1, 1994, to December 31, 2024. The searches were performed on February 1, 2025. The inclusion criteria are as follows: (1) Literature types are restricted to original research articles and review articles; (2) Only full-text publications will be considered. The exclusion criteria are outlined as follows: (1) Non-English publications; (2) Publication types encompass editorials, comments, case reports, letters, meeting abstracts, books, and retracted studies.

### 2.2 Search strategy and selection process

The search formula utilized in Web of Science is as follows: ((TS=rna-binding protein* OR TS=rna binding protein*) AND TS=osteosarcoma) AND (DT==(“ARTICLE” OR “REVIEW”) AND LA==(“ENGLISH”)). Publication Years: 1994-2024.

The search formula utilized in PubMed is as follows: (“rna binding proteins”[MeSH Terms] AND “osteosarcoma”[MeSH Terms]) AND ((1994/1/1:2024/12/31[pdat]) AND (english[Filter])). Article types include research articles, studies, and reviews.

The search formula utilized in Scopus is as follows: (TITLE-ABS-KEY (RNA binding protein) AND TITLE-ABS-KEY (Osteosarcoma)) AND PUBYEAR > 1993 AND PUBYEAR < 2025 AND (LIMIT-TO (DOCTYPE, “ar”) OR LIMIT-TO (DOCTYPE, “re”)) AND (LIMIT-TO (LANGUAGE, “English”)).

**Figure 1:**
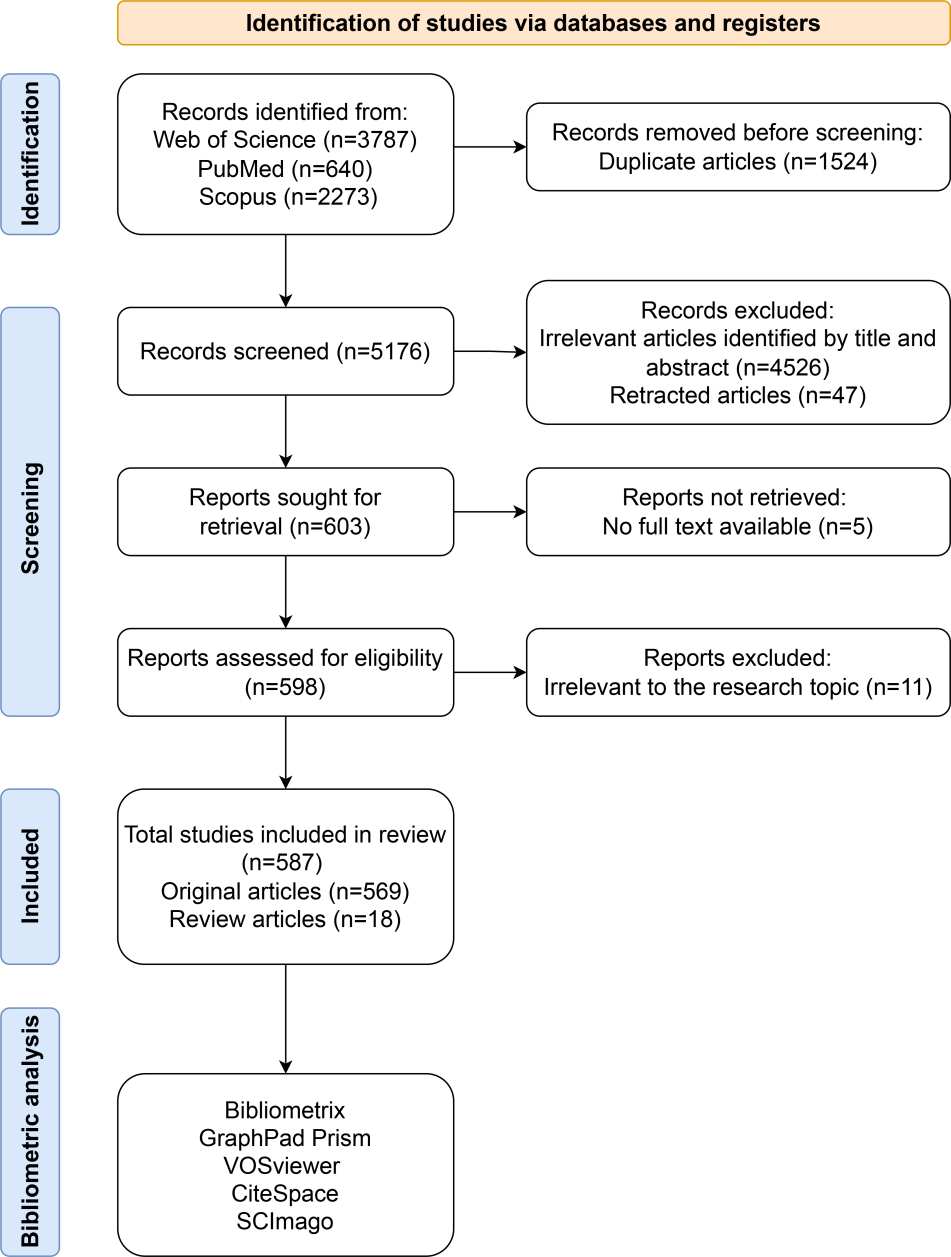
Flowchart illustrating the literature screening process.

Initially, a total of 6,700 articles were retrieved; however, two independent researchers (Zhiqian Gu & Songou Zhang) subsequently screened each article for relevance. During the literature screening process, any disagreements between the two researchers were resolved by a third researcher (Xudong Hu), who rendered the final decision. After excluding unrelated studies, a final selection of 587 articles (569 original articles and 18 review articles) pertinent to both RBPs and osteosarcoma was retained for bibliometric analysis. The detailed process of literature screening is illustrated in **Figure 1**.

### 2.3 Bibliometric analysis

The analysis utilized R bibliometrix and GraphPad Prism 10 for statistical evaluation; VOSviewer for visualizing co-occurrence relationships; CiteSpace for identifying key nodes and evolutionary paths; and SCImago for assessing influence. This multifaceted approach allowed for an in-depth examination of the current status, hotspots, and trends within this field.

## 3 Results

### 3.1 Annual of publications and citations

**Figure 2:**
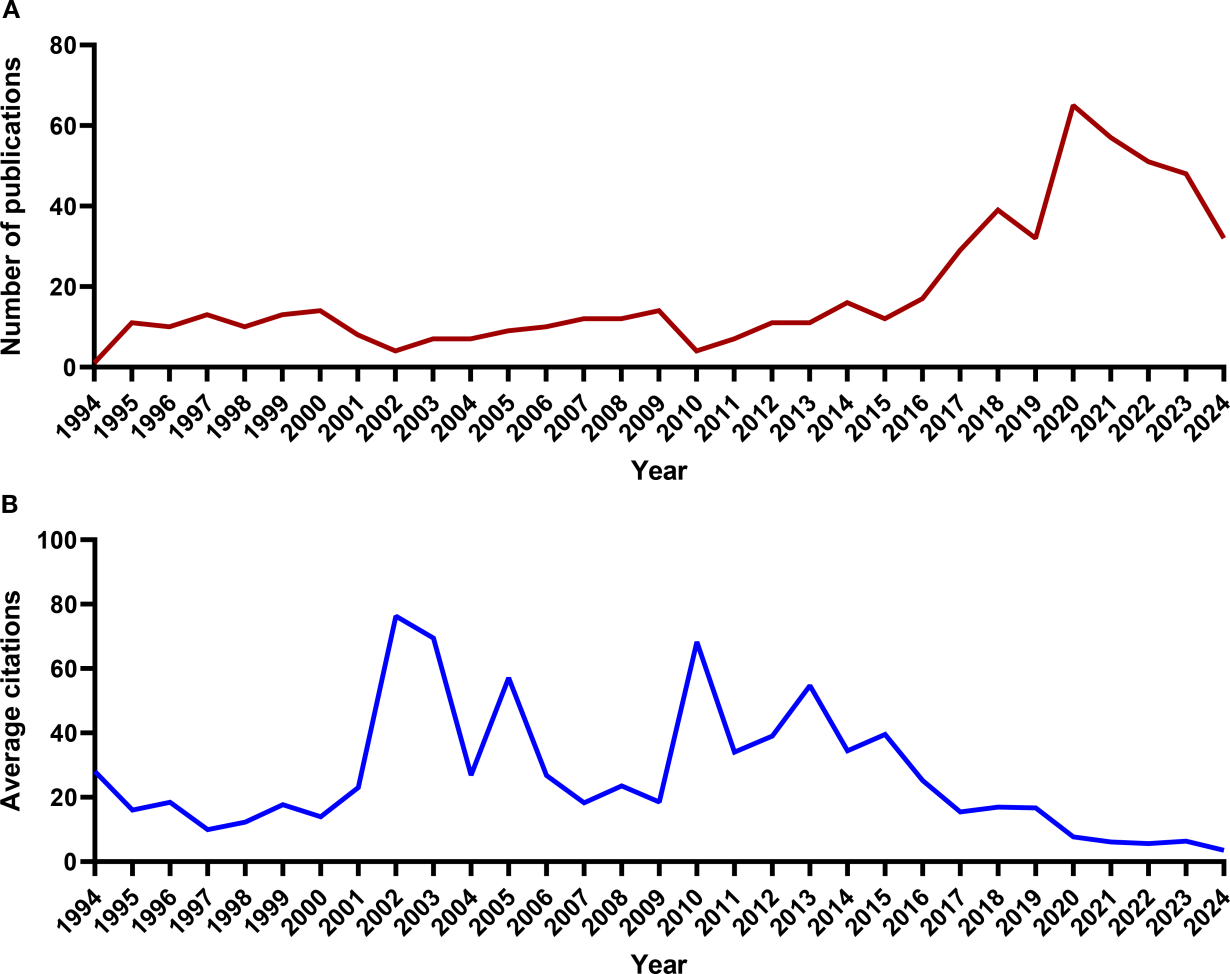
The trend of RBP publications in osteosarcoma from 1994 to 2024. **(A)** Annual publication counts. **(B)** Average citation counts per publication.

According to the analysis using the R Studio bibliometrix package, the publication time span for articles in this field ranges from 1994 to 2024, with a total of 587 articles (569 original articles and 18 review articles) published across 240 journals. The first research paper on this topic, published in 1994, focused on identifying Ewing’s sarcoma, osteosarcoma, and other small round cell sarcomas through the detection of EWS/FLI-1 (EWSR1) fusion transcripts (52). The study found that EWS/FLI-1 (EWSR1), as an RBP, was present in all Ewing’s sarcoma samples but absent in osteosarcoma, rhabdomyosarcoma, leiomyosarcoma, and malignant fibrous histiocytoma. The number of publications has steadily increased over the years, with a surge after 2016. The peak year for article publication was 2020, with 65 articles published that year (**Figure 2A**), indicating that this field has gained considerable attention in recent years. From the perspective of average citation counts in the literature, there were 5 citation peaks in 2002, 2003, 2005, 2010 and 2013 (**Figure 2B**), indicating that some important articles in this field were published during these years.

### 3.2 Country and organization analysis

**Figure 3:**
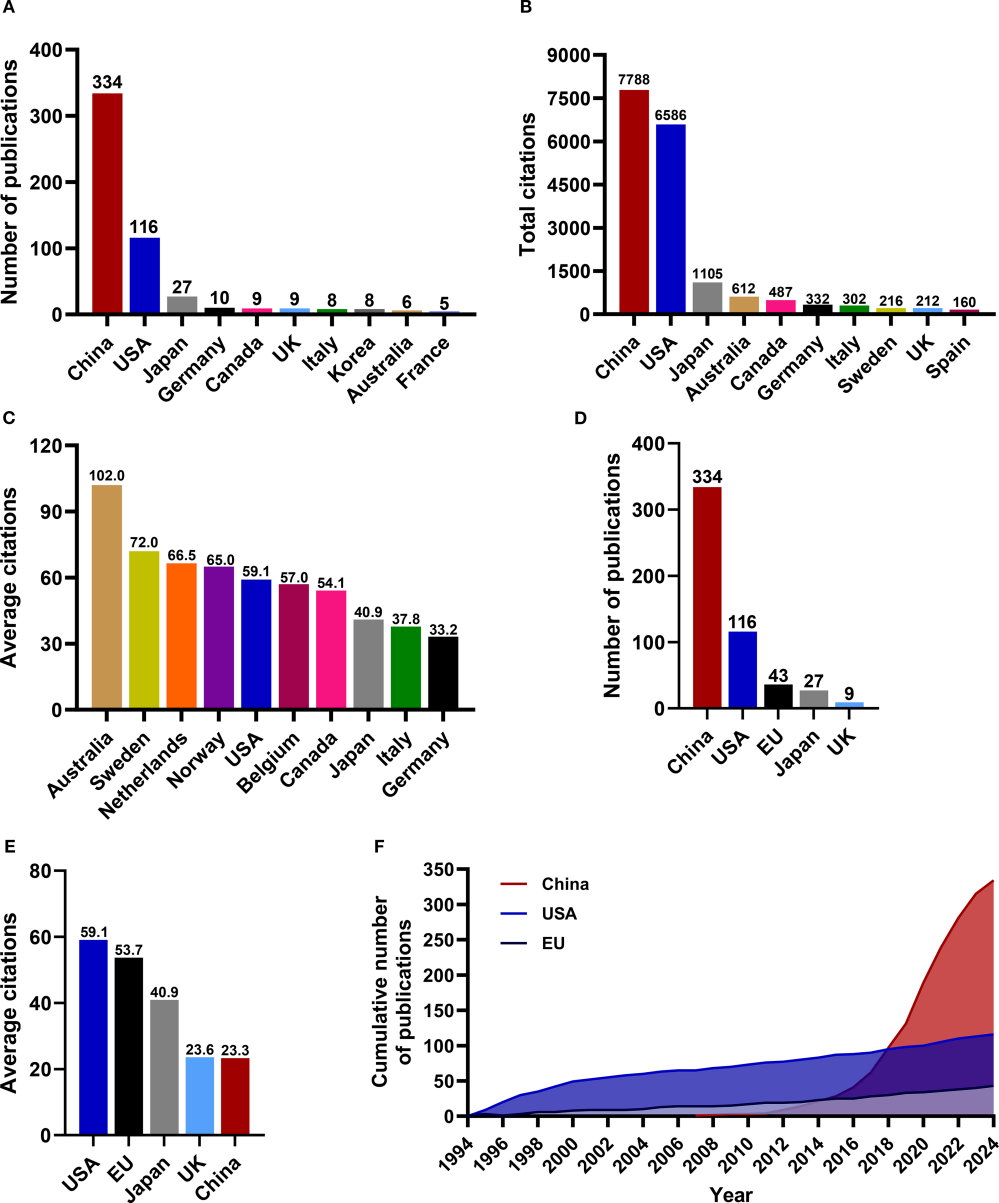
The landscape of research publications on RBPs in osteosarcoma across various countries. **(A)** The number of articles published by various countries. **(B)** The total citations attributed to each country. **(C)** The average citation counts among the various countries. **(D)** The volume of articles published in China, the USA, and the European Union. **(E)** Average citations in China, the USA, and the European Union. **(F)** The cumulative number of publications from China, the United States, and the European Union.

The analysis of countries and institutions can effectively highlight the active participants in this field and illustrate the dynamics of international cooperation. According to the affiliations of corresponding authors, a total of 30 countries contributed to research publications focused on RBPs in osteosarcoma. The top ten countries by publication volume included China, the United States, Japan, Germany, Canada, the United Kingdom, Italy, South Korea, Australia, and France; notably, China and the United States ranked as leaders in this regard (**Figure 3A**). The list of countries with the highest citation counts closely mirrored that of publication volume, with China, the United States, and Japan identified as the top three contributors (**Figure 3B**). However, when evaluated from an average citation perspective, China’s performance did not dominate. The average citation count is frequently a more accurate reflection of the influence exerted by a country, academic institution, or journal (53). The most recognized metric in this context is the journal impact factor. The nation leading by average citation volume were Australia, Sweden, and the Netherlands (**Figure 3C**). When comparing research achievements among China, the United States, and the European Union, it is evident that while China has produced the highest number of published papers (**Figure 3D**), its average number of citations remains the lowest (**Figure 3E**). Furthermore, China’s average citations are also lower than those of Japan and the United Kingdom, which raises concerns regarding the impact of its research contributions (**Figure 3E**). As illustrated in **Figure 3F**, both the European Union and the United States experienced a steady increase in publications from 1994 to 2024; conversely, China exhibited a remarkable surge since 2016. The findings indicate that over the past decade, China has witnessed a substantial rise in published papers within this field, ultimately surpassing both Europe and the United States. However, it continues to fall short concerning average citations count. Significant progress is necessary for China to enhance its academic influence on a global scale within this domain.

**Figure 4:**
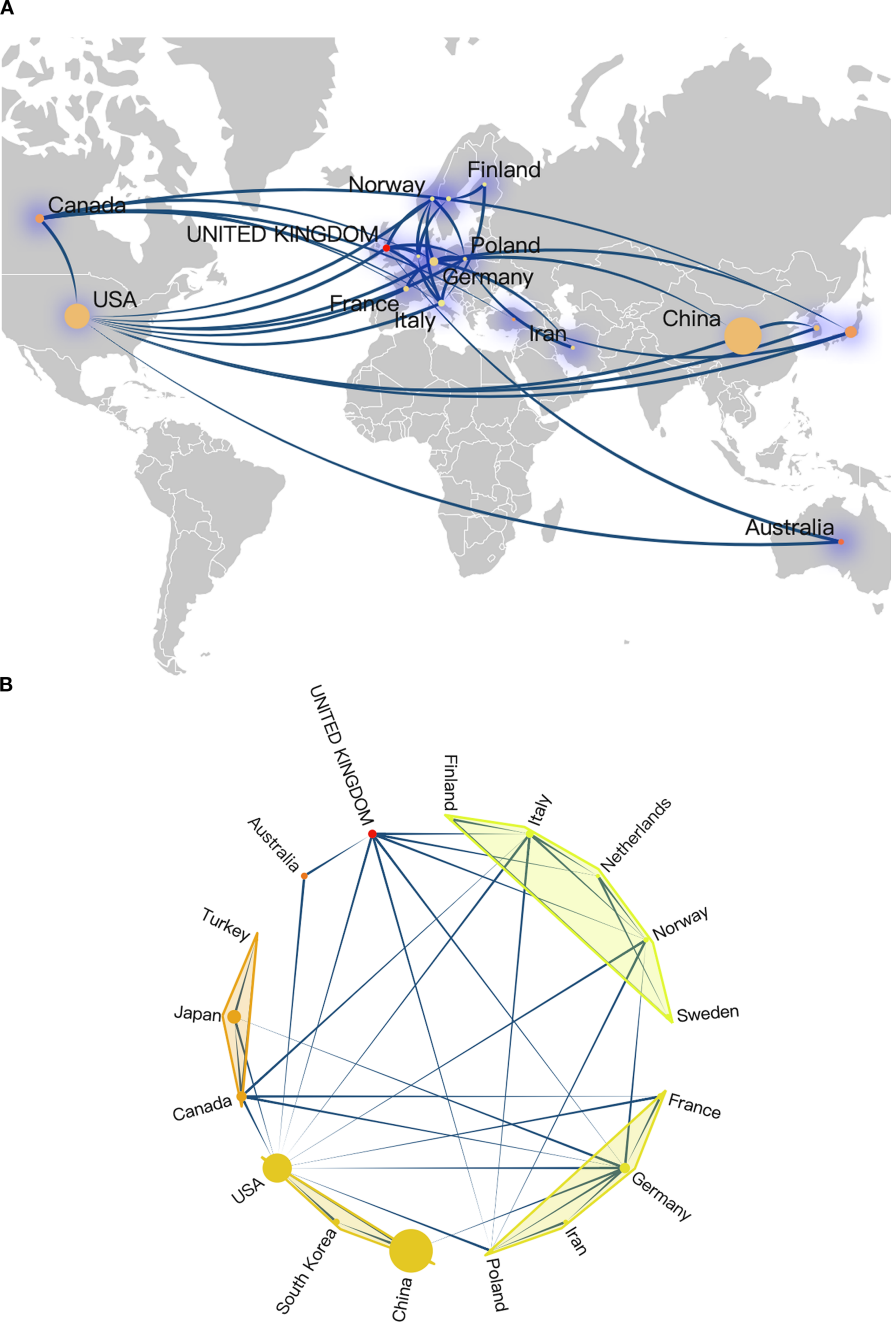
**(A, B)** National and regional collaborations on RBPs in osteosarcoma.

**Figures 4A, B** present an overview of the landscape of international collaboration. China primarily collaborated with researchers from the United States, Germany, and South Korea (**Figure 4B**). The United States maintained extensive collaborations with various European countries as well as Canada, Australia, and Japan (**Figure 4B**), underscoring its substantial global influence within this research domain. Researchers from several European countries, including the United Kingdom, Finland, Italy, Netherlands, Norway, Germany, and Poland, also engaged in frequent collaborations (**Figures 4A, B**).

The number of documents published by research institutions indicates their activity and research capacity in a specific field. In comparison, the average citation count better reflects the academic influence of these institutions (53). We identified 22 academic institutions that have published over ten papers, which include one hospital and the remainder being universities. **Table 1** outlines the ten most influential research institutions within this field. In the field of research on RBPs in osteosarcoma, Shanghai Jiao Tong University emerged as the leader with 21 publications. Central South University followed with 19 papers, while Nanjing Medical University contributed 18 publications. However, when evaluated based on the average citation count, the leading position was occupied by the University of Illinois in the United States. Among the top ten academic institutions ranked by average citation counts, four American research facilities held the first four slots, whereas six were from China. This trend underscores the significant academic impact exerted by American institutions in this area of research.

**Table 1:** Top 10 most productive institutions for RBPs in Osteosarcoma ranked by average citations (1994-2024).

Rank	Institution	Country	Average citations	Publications
1	University of Illinois	USA	120.8	10
2	Harvard University	USA	104.8	12
3	UTMD Anderson Cancer Center	USA	78.0	10
4	University Of Texas System	USA	76.5	12
5	Huazhong University of Science and Technology	China	46.9	16
6	Shanghai Jiao Tong University	China	39.1	21
7	Sun Yat Sen University	China	35.9	17
8	Zhejiang University	China	30.7	16
9	Central South University	China	27.8	19
10	Nanjing Medical University	China	25.3	18

**Figure 5:**
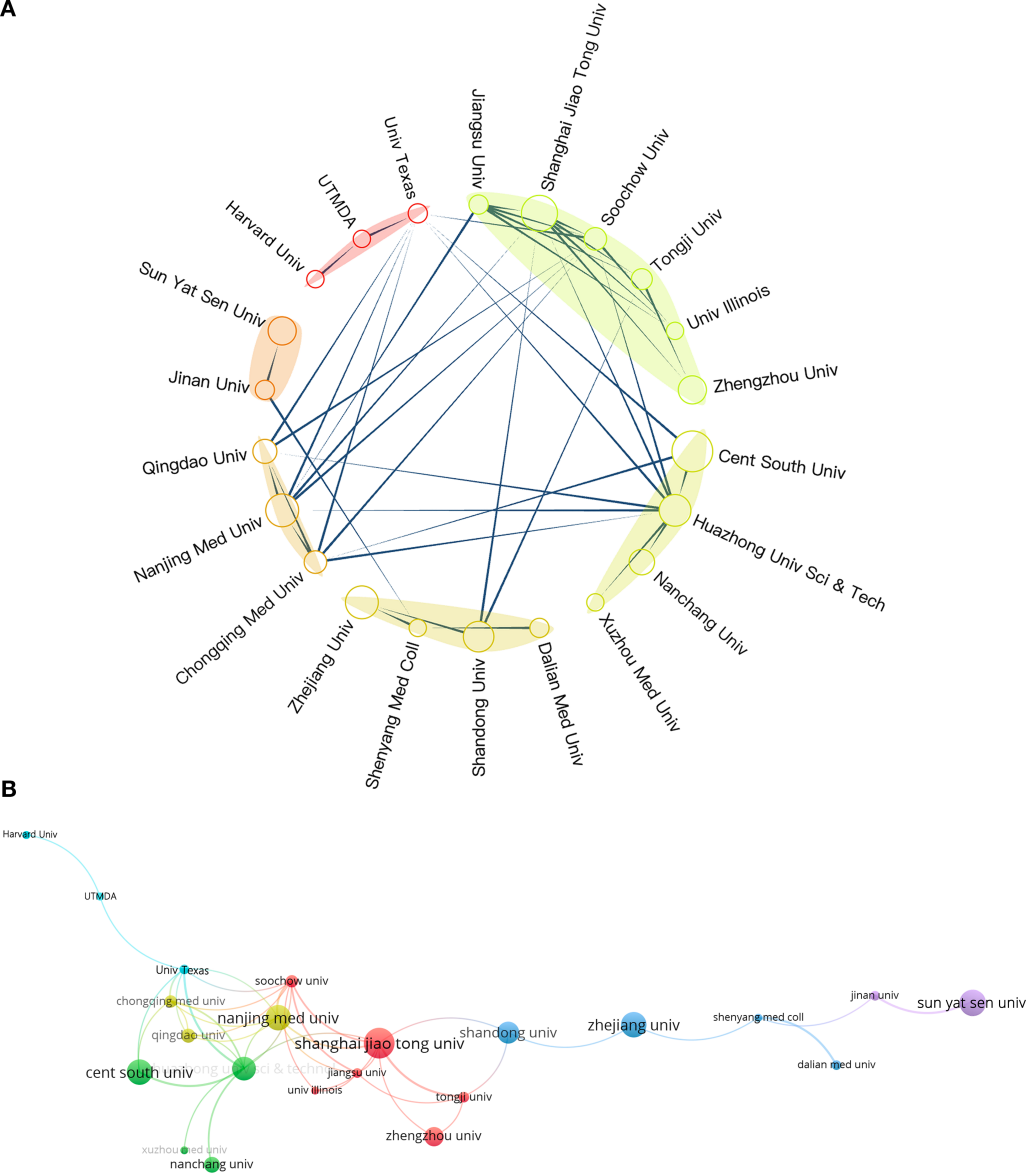
**(A, B)** Inter-institutional collaboration on RBPs in osteosarcoma.

We identified 22 institutions for an inter-institutional collaboration analysis. **Figures 5A, B** depict the collaboration network, where circle size indicates publication volume, lines represent collaborative relationships, and link thickness reflects collaboration intensity. The University of Texas System and the University of Illinois engaged in research collaborations with various universities in China. Cooperation among Chinese universities was widespread. The two universities that participated in collaborations with the highest number of institutions were Huazhong University of Science & Technology and Shanghai Jiao Tong University. This observation underscores their central role and significant influence within this research domain in China.

### 3.3 Author and journal analysis

**Figure 6:**
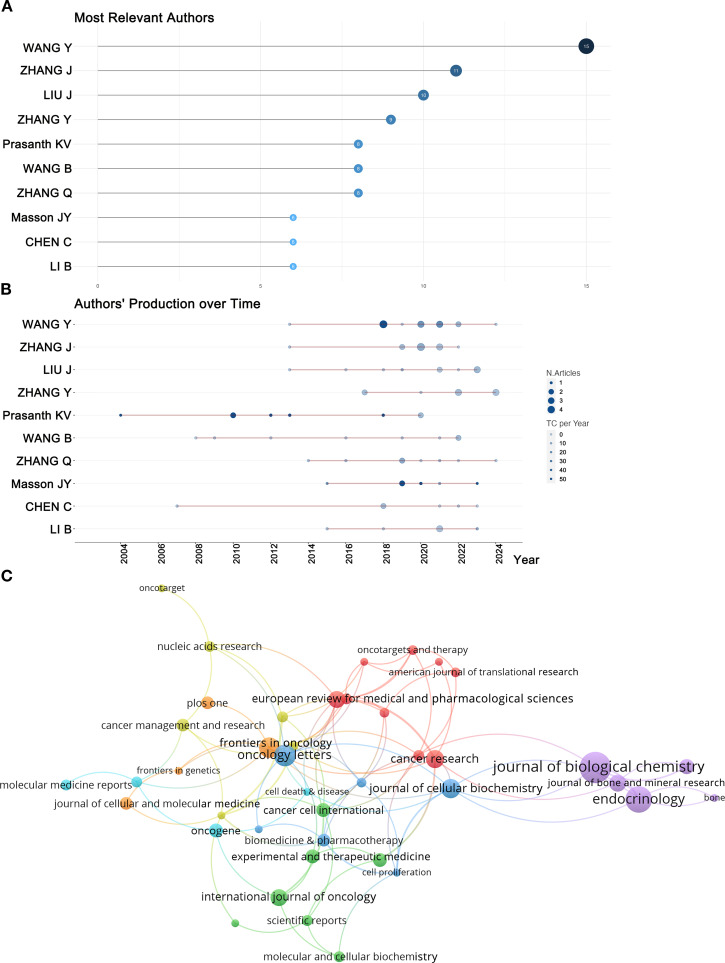
**(A)** The most relevant authors were identified based on their publication counts. **(B)** The most relevant authors’ production over time. **(C)** Co-citation network of journals.

Beyond examining the countries and institutions that have contributed to this field, analyzing the authors allows us to identify the most influential scholars and their research teams within this domain. **Figure 6A**, **Table 2** present the top ten most relevant authors on RBPs in osteosarcoma based on their publication counts. Among these leading authors, eight were from China, while one was from the United States and another from Canada. This highlights the significant contributions of these three countries in this field. The top three researchers ranked by the number of article publications were all affiliated with institutions in China. However, when assessed based on the H-index—an indicator of citation impact—only one of these prominent researchers originated from China. Notably, Jean-Yves Masson from Laval University (Canada) achieved the highest H-index, significantly surpassing Kannanganattu V. Prasanth from the University of Illinois (USA), who ranked second, followed by Yang Wang from Harbin Medical University (China) in third place.

**Table 2:** Top 10 most relevant authors and their H-index impact on RBPs in osteosarcoma.

Rank	Author	H-index	Affiliation	Country	Publications
1	Wang Y	16	Harbin Med Univ	China	15
2	Zhang J	14	Nanchang Univ	China	11
3	Liu J	12	Sun Yat Sen Univ	China	10
4	Zhang Y	12	Chinese Acad Sci	China	9
5	Prasanth KV	33	University of Illinois	USA	8
6	Wang B	15	Shandong Univ	China	8
7	Zhang Q	8	Nanjing Med Univ	China	8
8	Masson JY	50	Laval University	Canada	6
9	Chen C	10	Hubei Univ Med	China	6
10	Li B	13	Shandong Univ	China	6

**Figure 6B** shows the publication trends and citation counts of these prolific authors over the years. Nodes represent an author’s publishing activity in a specific year: larger nodes indicate more published papers, while darker colors denote higher citation frequencies. The red line represents the timeline of the author’s research outputs. In terms of total citations for published papers, Professor Kannanganattu V. Prasanth from the United States ranked first, while Professor Jean-Yves Masson from Canada Occupied the second position. The cumulative citation counts for these two authors significantly exceed those of their peers. Professor Prasanth’s notable publications were released in 2004, 2010, 2012, and 2013; each paper published during these years received over 100 citations. In contrast, Professor Masson published influential works in 2019, 2020, and 2023; similarly, each of his contributions from these years garnered more than 50 citations. Collectively, these two professors have made substantial contributions to the field of research on RBPs in osteosarcoma.

Journal co-citation analysis serves as an essential methodology for identifying influential journals within specific research domains. We focused on journals that published more than four articles, as illustrated in **Figure 6C**. The size of each node reflects the total number of publications; larger nodes indicate higher publication volume. The connections between nodes indicate the intensity of collaboration or citation, with thicker lines denoting stronger associations. The co-cited journals were categorized into seven distinct clusters, represented by various colors. Notably, the *Journal of Biological Chemistry* exhibited the highest co-citation count at 1,679 and published the most articles in this field, with a total of 22 relevant publications (**Table 3**). The top five journals ranked by co-citation frequency are as follows: *Journal of Biological Chemistry* (1,679), *Cancer Research* (979), *Endocrinology* (870), *Journal of Bone and Mineral Research* (699), and *Nucleic Acids Research* (660). These findings highlight their significant academic influence within the discipline.

**Table 3:** Top 10 high-output journals.

Rank	Journal	Country	JIF(2025)	JCR	Publications
1	Journal of Biological Chemistry	USA	3.9	Q2	22
2	Endocrinology	USA	3.3	Q2	18
3	Oncology Letters	Greece	2.2	Q3	14
4	Frontiers in Oncology	USA	2.8	Q2	13
5	Journal of Cellular Biochemistry	USA	2.8	Q3	12
6	Cancer Research	USA	16.6	Q1	11
7	International Journal of Oncology	Greece	4.9	Q1	10
8	Journal of Bone and Mineral Research	USA	5.9	Q1	9
9	Oncogene	England	7.3	Q1	8
10	Cancer Cell International	England	6	Q1	8

**Table 3** lists the top ten journals by publication volume, along with their impact factors and JCR quartiles for 2025. The impact factors from the Journal Citation Reports 2025 database serve as key indicators of journal influence and credibility. Among the top ten journals, six originated from the United States, two from England, and two from Greece. The highest impact factor belonged to *Cancer Research* (USA) at 16.6 with a JCR classification of Q1. *Oncogene* (England) ranked second with an impact factor of 7.3 and held a Q1 classification. In third place was *Cancer Cell International* (England), boasting an impact factor of 6 and a Q1 classification as well. Fourth was the *Journal of Bone and Mineral Research* (USA) with an impact factor of 5.9, maintaining a Q1 rating. These rankings highlight the significant influence of both the United States and the United Kingdom in this research field.

### 3.4 Publications with the highest citation frequency

Highly cited publications in scientific fields are regarded as exemplary works that signify scientific excellence (54). Analyzing highly cited publications provides us with a more precise comprehension of the foundational and core content within the field. **Table 4** presents the top ten publications with the highest citation counts in this domain, ranked according to total citations from 2006 to 2020. The most frequently cited article in this research field was a review titled “*The potential role of RNA N6-methyladenosine in Cancer progression RNA N6*” by Professor Shaoqing Ju from China (2020), which received 757 citations. This study summarized recent significant advancements, highlighting that METTL3 promotes osteosarcoma cell proliferation by regulating LEF1 m6A methylation and activating the WNT/β-catenin signaling pathway.

**Table 4:** Top 10 most cited publications on RBPs in osteosarcoma.

Rank	Title	Type	Corresponding author	Journal	Year	Citation	Major themes
1	The potential role of RNA N6-methyladenosine in Cancer progression RNA N6	Review	Shaoqing Ju	Molecular Cancer	2020	757	METTL3 promotes osteosarcoma cell proliferation by regulating LEF1 m6A methylation and activating the WNT/β-catenin pathway
2	Long Noncoding RNA MALAT1 Controls Cell Cycle Progression by Regulating the Expression of Oncogenic Transcription Factor B-MYB	Article	Kannanganattu V. Prasanth	PLOS Genetics	2013	698	Decreased MALAT1 expression increases SRSF1 binding to pre-mRNAs (B-MYB and CENPE), thereby regulating alternative splicing in osteosarcoma cells
3	Structural determinants of the cellular localization and shuttling of TDP-43	Article	Francisco E. Baralle	Journal of Cell Science	2008	570	TDP-43 (TARDBP) shuttles between the nucleus and cytoplasm of osteosarcoma cells in a transcription-dependent manner
4	Competing Protein-RNA Interaction Networks Control Multiphase Intracellular Organization	Article	Clifford P. Brangwynne	Cell	2020	541	G3BP binds polyA+ mRNA and associates with stress granules in U2OS cells
5	Control of c-myc mRNA stability by IGF2BP1-associated cytoplasmic RNPs	Article	Stefan Hüttelmaier	RNA	2009	313	GF2BP1 stabilizes c-myc mRNA in U2OS cells by binding to CRD
6	Eukaryotic initiation factor 2α-independent pathway of stress granule induction by the natural product pateamine A	Article	Jun O. Liu	Journal of Biological Chemistry	2006	265	Phosphorylated eIF2α recruits to mRNA via the eIF4F complex (eIF4E, eIF4G, and eIF4A), facilitating stress granule formation in U2OS cells
7	Translational Activation of HIF1α by YB-1 Promotes Sarcoma Metastasis	Article	Poul H. Sorensen	Cancer Cell	2015	239	YB-1 (YBX1) interacts with HIF1A mRNA to enhance its translation, promoting osteosarcoma progression
8	YB-1 regulates stress granule formation and tumor progression by translationally activating G3BP1	Article	Poul H. Sorensen	Journal of Cell Biology	2015	236	YB-1 (YBX1) interacts with G3BP1 mRNA to enhance its translation, promoting osteosarcoma progression
9	TDP-43 regulates retinoblastoma protein phosphorylation through the repression of cyclin-dependent kinase 6 expression	Article	Francisco E. Baralle	Proceedings of the National Academy of Sciences	2008	228	TDP-43 (TARDBP) inhibits Cdk6 expression by binding to the (GU)n repeats of its pre-mRNA in U2OS cells
10	The Mitochondrial RNA-Binding Protein GRSF1 Localizes to RNA Granules and Is Required for Posttranscriptional Mitochondrial Gene Expression	Article	Eric A. Shoubridge	Cell Metabolism	2013	203	ND6 mRNA is enriched in GRSF1 immunoprecipitates and crucial for post-transcriptional mitochondrial gene expression in 143B osteosarcoma cells

The second most cited publication was authored by Professor Kannanganattu V. Prasanth from the United States, entitled: “*Long Noncoding RNA MALAT1 Controls Cell Cycle Progression by Regulating Oncogenic Transcription Factor B-MYB Expression*”. This study primarily demonstrated that reduced expression of MALAT1 leads to an increased binding affinity between SRSF1 and pre-mRNA (B-MYB and CENPE), thereby regulating alternative splicing mechanisms in osteosarcoma cells.

Among the top 10 most cited publications, research papers ranked 3rd and 9th were authored by Professor Francisco E. Baralle from the International Centre for Genetic Engineering and Biotechnology (ICGEB) in Trieste, Italy. Meanwhile, those ranked 7th and 8th were contributed by Professor Poul H. Sorensen of the University of British Columbia in Canada. This highlights the significant impact exerted by both professors within this field.

### 3.5 Analysis of the most active topics

#### 3.5.1 Subject category bursts

**Figure 7:**
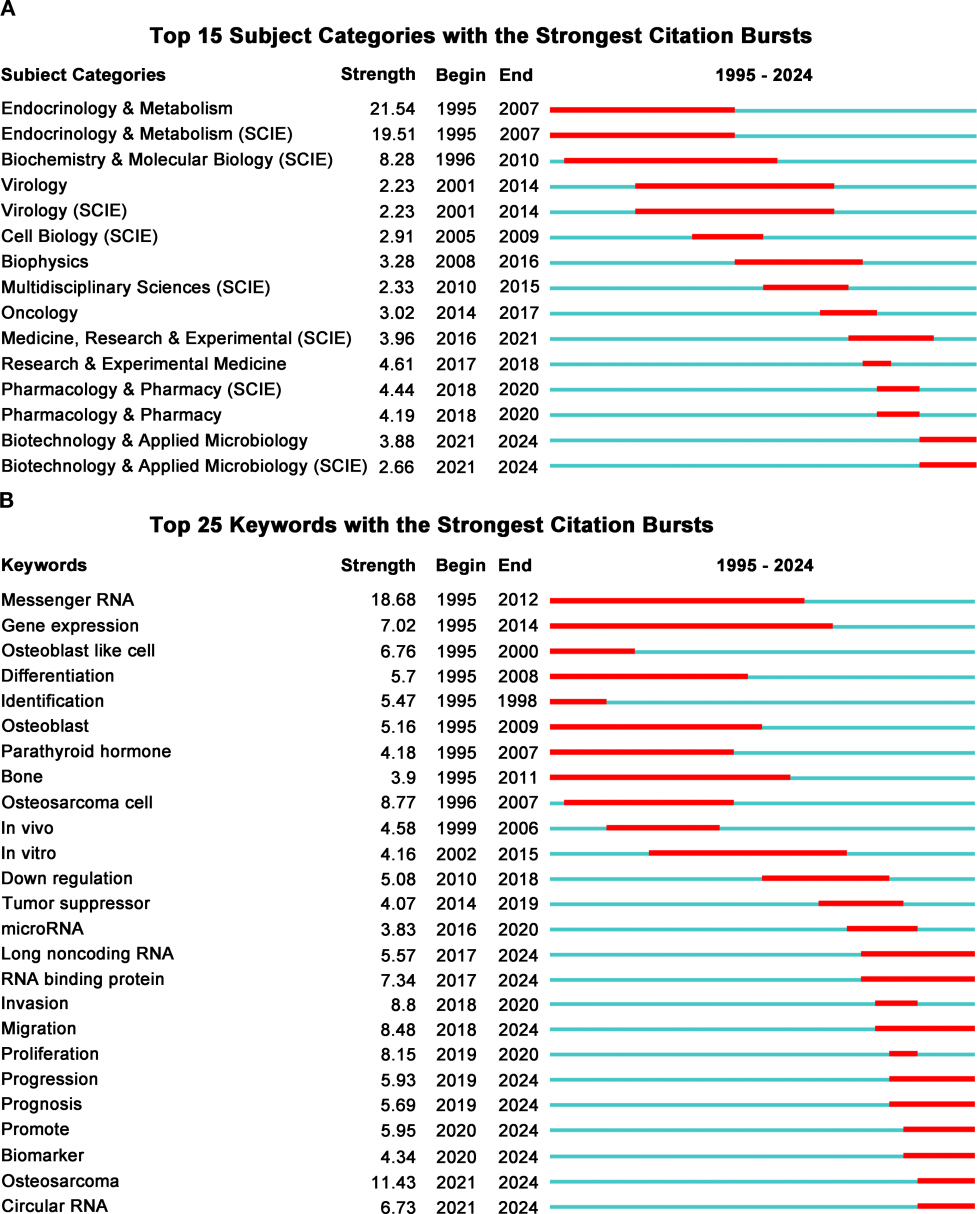
**(A)** Top 15 subject categories with the strongest citation bursts. **(B)** Top 25 keywords with the strongest citation bursts.

Subject category burst analysis reveals research hotspots and trends, enhancing our understanding of the development in this field. **Figure 7A** displays the top 15 subject categories with bursts. The left side categorizes various disciplines, while the right indicates the year each burst occurred. The red line shows burst duration, and the blue line represents the overall time span.

The findings indicate that during the initial research phase in this field (1995 to 2010), scholarly attention primarily centered on areas such as endocrinology, metabolism, virology, and cell biology. In the subsequent mid-stage of research (2010 to 2018), there was a gradual shift toward disciplines including biophysics and oncology. In recent years (2018 to 2024), research efforts have increasingly concentrated on fields such as pharmacology, biotechnology, and medical science. This transformation highlights the evolving nature of research methodologies and focuses. Early investigations primarily concentrated on elucidating the role of RBPs in osteosarcoma, whereas contemporary research hotspots have shifted towards identifying novel therapeutic targets and developing new pharmacological agents.

#### 3.5.2 Keyword bursts

Similarly, by analyzing the keywords of publications in this field, we can quickly identify its research hotspots and development trends. A more comprehensive analysis employing keyword bursts was conducted to examine research trends concerning RBPs in osteosarcoma (**Figure 7B**). During the early stages of investigation from 1995 to 2008, focal points included keywords such as mRNA and gene expression. This indicates that preliminary studies primarily centered on elucidating the expression patterns of various genes within this domain. In recent years (2018 to 2024), there has been a significant shift in keyword bursts towards cellular phenotypes, specifically proliferation, migration, and invasion. This transition underscores that current research increasingly clarifies the role of RBPs in osteosarcoma through experiments focused on cell phenotypes. Furthermore, recent years (2016 to 2024) have witnessed the emergence of keywords such as circular RNA, microRNA, and long non-coding RNA. These developments reflect a significant transition in emphasis from coding RNA toward non-coding RNA.

#### 3.5.3 Keyword clustering analysis

**Figure 8:**
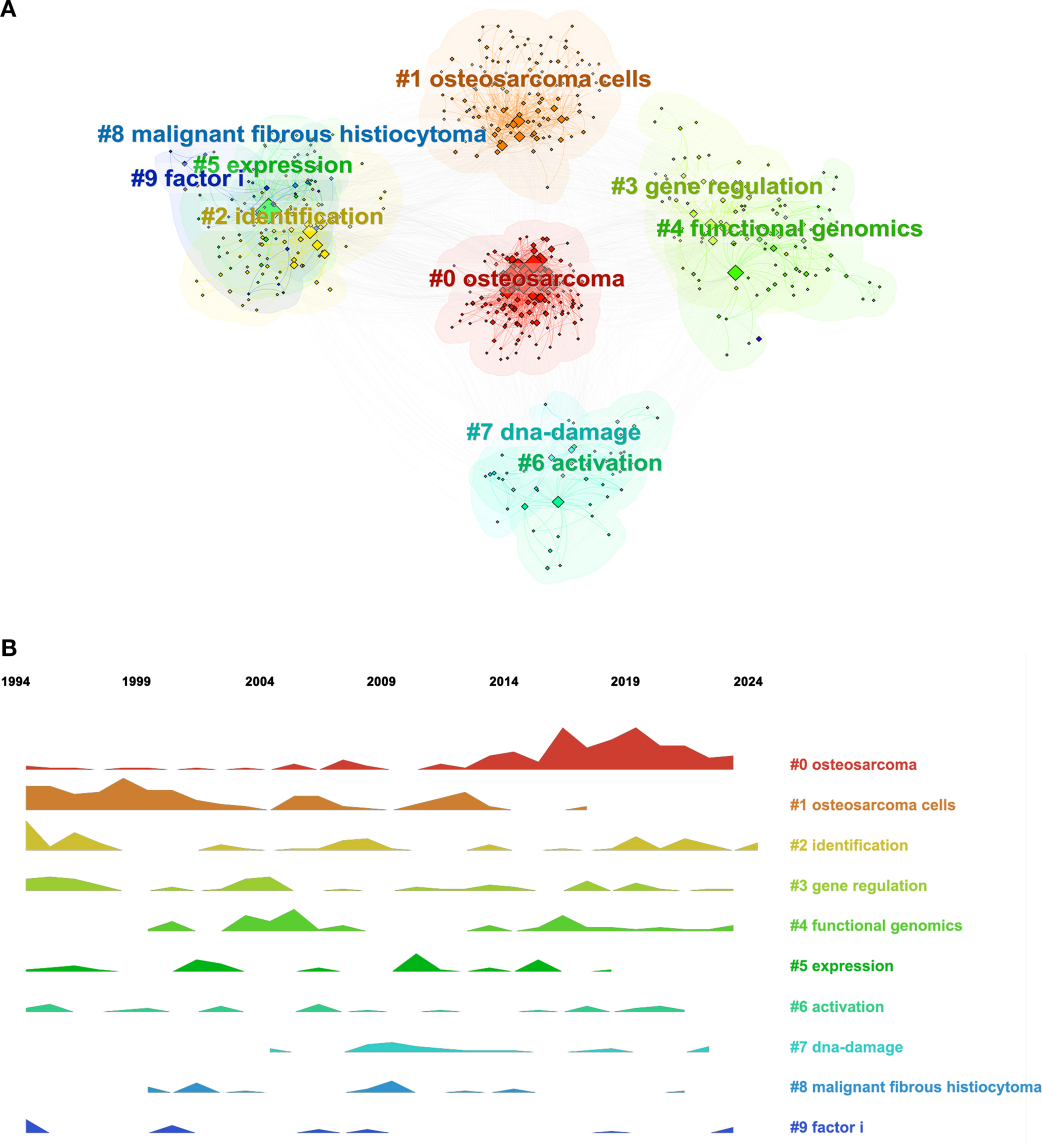
Analysis of keywords in RBPs research on osteosarcoma. **(A)** Keyword clustering map. **(B)** Ridgeline plot of research topic evolution over time.

Further analysis indicated that the keywords in this domain exhibited strong intrinsic correlations, with specific keywords forming distinct clusters based on their affinities. Identifying these clusters offers a more intuitive understanding of the various subfields within RBPs research related to osteosarcoma. As illustrated in **Figure 8A**, the keyword clusters can be categorized into four primary groups: osteosarcoma disease (#0: osteosarcoma, #1: osteosarcoma cells, #8: malignant fibrous histiocytoma), RBP functional research (#3: gene regulation, #4: functional genomics, #7: DNA damage), and different types of RBP research (#2: identification, #9: factor I, #5: expression). The timeline map of keywords illustrates the distribution and frequency of the top ten clustering keywords from 1994 to 2024, highlighting the evolving research themes over time (**Figure 8B**). **Figure 8B** illustrates that from 2016 to 2024, research on RBPs in osteosarcoma primarily centered on gene identification, gene regulation, and functional genomics.

## 4 Discussion

### 4.1 General information

RBPs have become a prominent area of research in tumor biology in recent years. The study of RBPs in osteosarcoma began in 1994 and peaked around 2020. From 1994 to 2015, the annual number of published papers in this field remained stable, with just one paper published in 1994 and no more than 20 each subsequent year. However, from 2016 to 2024, research output grew significantly, peaking at 65 papers in 2020 (**Figure 2A**). China and the United States emerged as the leading countries in terms of the volume of research on RBPs in osteosarcoma. The three universities that made significant contributions to this field were all located in the United States: the University of Illinois, Harvard University, and UT MD Anderson Cancer Center. In China, the two leading institutions in this research domain were Huazhong University of Science & Technology and Shanghai Jiao Tong University. The scholars who made significant contributions to this field included Kannanganattu V. Prasanth (USA), Jean-Yves Masson (Canada), and Yang Wang (China). Additionally, the analysis of subject categories and keywords indicates that 2018 represented a pivotal turning point in the research trajectory within this field. Prior to 2018, investigations primarily focused on elucidating the molecular roles and regulatory mechanisms associated with specific RBPs in osteosarcoma. Conversely, post-2018 studies have demonstrated a pronounced shift toward exploring how RBPs influence the tumor microenvironment as well as their role in epigenetic regulation.

### 4.2 Evolving research paradigms

**Figure 9:**
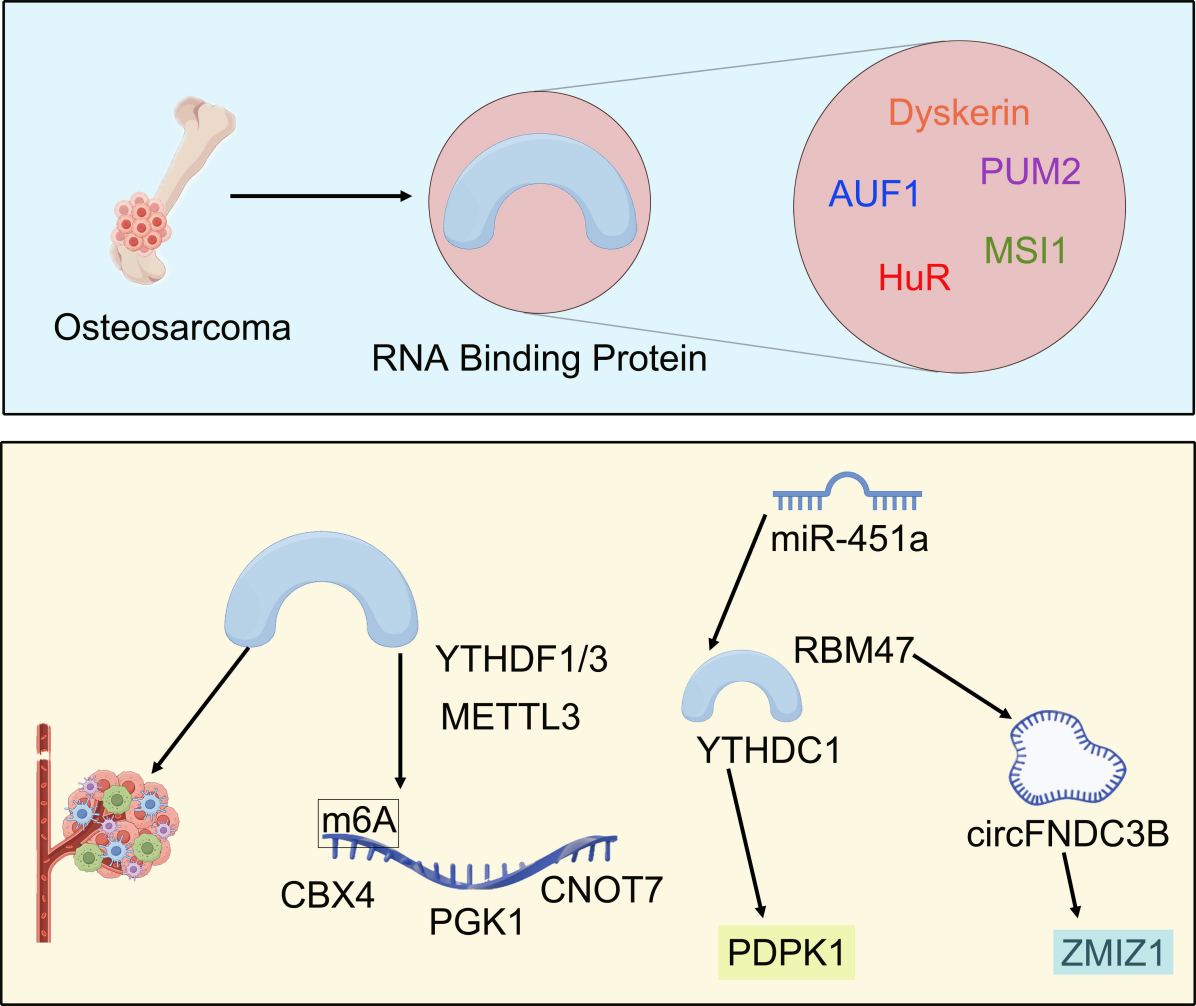
The evolution of research paradigms for RBPs in osteosarcoma.

Further comprehensive analysis of the literature and bibliometric data in this research field has concluded that the area of RBPs in osteosarcoma research exhibits a phased shift in focus (**Figure 9**). Prior to 2018, investigations primarily concentrated on an in-depth exploration of the direct regulatory mechanisms by specific RBPs on the core biological functions of osteosarcoma cells, thereby establishing a molecular foundation for understanding RBPs roles in this disease.

Previous studies have demonstrated that multiple key RBPs significantly influence the malignant biological behaviors of osteosarcoma cells through direct regulation of gene expression networks. At the level of mRNA stability regulation, HuR is recruited by oncogenic long non-coding RNA B4GALT1-AS1 to specifically bind and stabilize YAP mRNA, thereby promoting tumor stem cell characteristics, migration, and chemotherapy resistance (55). AUF1 further enhances cell invasion and proliferation by concurrently stabilizing the mRNAs associated with epithelial-mesenchymal transition inducer ZEB1 and AKT signaling pathway activator PDK1; its activity can be inhibited by tumor suppressor microRNAs miR-141/146b-5p (56). MSI1 disrupts G1/S phase arrest by binding to the 3’ untranslated region (UTR) of cell cycle inhibitors p21/p27, inhibiting their translational expression and leading to unregulated cell cycling (22).

At the level of competitive RNA interactions, PUM2 functions as a tumor suppressor by competing with pro-oncogenic miRNAs miR-590-3p/miR-9 for binding at the 3’ UTR of STARD13 mRNA. This interaction blocks miRNA-mediated degradation, preserving PUM2’s function as a tumor suppressor and consequently inhibiting cell proliferation, migration, and maintenance of stemness characteristics (21), underscoring RBPs’ value as regulatory hubs or “molecular switches”.

Furthermore, regarding cell fate determination, the absence of Dyskerin can induce cellular senescence via telomere-independent pathways, while also enhancing the cells’ anti-apoptotic capacity in response to genotoxic stress. Notably, this process can be reversed through the application of epigenetic inhibitors such as histone deacetylase inhibitors (57). Moreover, the overexpression of the oncogene NOVA1 directly promotes cell viability, clone formation, and invasive capacity; conversely, its knockout significantly suppresses these malignant phenotypes (58). In summary, these RBPs target transcription factors including YAP and ZEB1 as well as cell cycle inhibitors p21/p27 and AKT kinase pathways. This interaction creates a cascading network that links molecular stability regulation with malignant phenotype and treatment resistance. Such findings elucidate the critical biological roles of RBPs in osteosarcoma progression and provide a molecular foundation for advancing this field from basic mechanisms toward clinical applications.

Since approximately 2018, research focus has significantly expanded and deepened, revealing two prominent emerging trends. Firstly, there is an in-depth exploration of the role of RBPs in shaping the tumor microenvironment and mediating epigenetic regulation. At the level of the immune microenvironment, a prognostic model constructed from seven core RBPs effectively distinguishes the immune characteristics of osteosarcoma patients (19). The low-risk group exhibits markedly elevated immune scores, matrix scores, and levels of immune cell infiltration, along with high expression of immune checkpoint molecules such as PD-1 and CTLA-4—indicating an active immune response. Conversely, the high-risk group demonstrates an immunosuppressive microenvironment characterized by increased tumor purity and reduced immune cell infiltration conditions alongside poor prognosis. This suggests that RBPs influence the efficacy of immunotherapy responses through overall regulation of immune cell recruitment and checkpoint expression.

At the level of epigenetic regulation, m6A modification-related RBPs form a cascading regulatory network: methyltransferase METTL3 enhances m6A modification on CBX4 mRNA—the oncogene—thereby improving its stability which leads to upregulation of matrix metalloproteinases MMP2/MMP9 as well as mesenchymal marker N-Cadherin promoting tumor metastasis (59). In parallel, YTHDF3—a reader protein for m6A—directly drives aerobic glycolysis in osteosarcoma cells while accelerating tumor proliferation by recognizing and stabilizing PGK1 mRNA at specific m6A sites; this key glycolytic enzyme plays a pivotal role in metabolism (60). Furthermore, during this process, YTHDF1 promotes CNOT7 mRNA expression by binding to its corresponding m6A modification ultimately activating invasive phenotypes associated with tumors (61). In summary, RBPs facilitate malignant evolution through two synergistic mechanisms: they shape an immunosuppressive microenvironment to evade immune surveillance while also stabilizing oncogenic transcripts via their central role in m6A modifications.

Second, we concentrated on analyzing the intricate interaction network between RBPs and various non-coding RNAs. In osteosarcoma, RBPs and non-coding RNAs establish a bidirectional synergistic regulatory network that collectively drives the malignant process. miR-451a inhibits YTHDC1-mediated m6A modification, thereby destabilizing its downstream target PDPK1 mRNA and ultimately impairing the activation of the AKT/mTOR pathway as well as the epithelial-mesenchymal transition (EMT) process (62). This creates a “miRNA-RBP-m6A” cross-regulatory axis.

At the level of non-coding RNA generation, RNA-binding motif protein 47 (RBM47) specifically interacts with an intronic sequence adjacent to circFNDC3B, facilitating its circularization. This interaction leads to a decrease in the abundance of linear FNDC3B mRNA—an oncogene—while promoting the formation of circFNDC3B, which possesses tumor-suppressive properties (63). Conversely, long non-coding RNA ZMIZ1-AS1 directly recruits polypyrimidine tract binding protein 1 (PTBP1), binds to the 3’ UTR of its homologous gene ZMIZ1 mRNA, and enhances its stability. As a result, this process amplifies the expression levels of this oncogenic transcript (64).

From a functional regulation perspective, the newly generated circFNDC3B acts reversely as a molecular bait that competitively binds to IGF2BP1. This binding impedes IGF2BP1’s stabilizing effect on FNDC3B mRNA, resulting in a regulatory mechanism characterized by the cycle of “RBP promotes cyclization – circular RNA inhibits RBP function – target gene expression imbalance” (63). This bidirectional interaction ultimately converges into two core oncogenic axes: one is the RBM47-circFNDC3B-IGF2BP1 imbalance axis, which impedes tumor progression through dual inhibition of FNDC3B expression—achieved by reducing linear transcripts via circularization and competing to block mRNA stability; while the other is the ZMIZ1-AS1-PTBP1-ZMIZ1 stabilization axis that enhances transcript stability for homologous genes via lncRNA-RBP complexes and activates downstream signals associated with proliferation and invasion. The study revealed that these two mechanisms correspond to key malignant phenotypes of osteosarcoma: dysregulation of FNDC3B directly promotes epithelial-mesenchymal transition (EMT) and metastatic potential, whereas overexpression of ZMIZ1 drives uncontrolled cell cycle dynamics and resistance to apoptosis. Furthermore, PTBP1-mediated stabilization of ZMIZ1 has been shown to significantly accelerate tumor growth *in vivo* (64). These findings collectively underscore that RBPs serve not only as initiators of non-coding RNA generation (such as RBM47-driven circularization), but also as execution hubs for non-coding RNA function. Moreover, non-coding RNAs dynamically modulate RBP activity through negative feedback regulation or spatial recruitment, thereby providing novel avenues for targeted intervention.

This transition from research focused on individual target functions to the regulation of microenvironments and network interactions represents a gradual evolution rather than a sudden change. It reflects the progress of the field toward a more integrated approach with substantial clinical potential, offering an expanded perspective for the development of innovative diagnostic and therapeutic strategies for osteosarcoma.

### 4.3 Future directions

#### 4.3.1 Mechanistic depth: linking RBP dysregulation to osteosarcoma heterogeneity using multi-omics technologies

Integrating multi-omics technologies, such as genomics, transcriptomics, proteomics, and epigenomics, is essential for understanding the complex relationship between RBP dysregulation and osteosarcoma heterogeneity. By comprehensively analyzing genomic alterations in RBP genes, transcriptomic profiles of RBPs and their target RNAs, proteomic changes in RBP expression and activity, and epigenetic modifications associated with RBP function, we can gain deeper insights into the molecular mechanisms driving osteosarcoma occurrence and development. This approach will not only enhance our understanding of the disease but also help identify potential new therapeutic targets (65, 66).

#### 4.3.2 Therapeutic innovation: development of therapies targeting RBPs

With increasing recognition of RBPs’ key role in osteosarcoma, targeted therapies against these proteins hold great promise. Designing small molecule inhibitors that disrupt RBP-mRNA interactions could potentially inhibit osteosarcoma cell growth. In addition, other strategies such as antisense oligonucleotides or RNA interference could be explored to modulate RBP activity. However, challenges such as effective drug delivery and minimizing off-target effects must be carefully addressed to ensure both the safety and efficacy of these therapies. Clinical integration: Validation of RBPs as biomarkers for early diagnosis or treatment response. The identification and validation of RBPs as reliable biomarkers for osteosarcoma could transform clinical practice. By detecting specific RBPs or their associated molecular signatures in patient samples, early diagnosis of osteosarcoma could be achieved, enabling timely intervention and improving patient outcomes. In addition, monitoring changes in RBP levels throughout treatment may provide valuable insights into treatment responses, facilitating personalized treatment plans. Large-scale clinical studies using well-defined cohorts and standardized detection methods will be essential for establishing the clinical utility of RBPs as biomarkers.

### 4.4 Limitations

Based on current search results, this study is the first bibliometric assessment of RBPs in osteosarcoma. However, several limitations should be acknowledged. First, the literature sources primarily include English publications. While English is a key medium for academic communication, this focus excludes relevant studies published in other languages, potentially introducing regional bias and limiting a full understanding of the global research landscape. Second, some papers may not have been included due to differing focuses (e.g., certain RNA-binding proteins also acting as DNA-binding proteins) or outdated terminology for specific RBPs. Although these studies are relevant, they may not explicitly mention “RNA-binding protein” or use commonly recognized nomenclature in their titles or abstracts; instead, they might refer to older terms that are less widely used. These overlooked studies could hold significant value; however, expanding search parameters requires careful review of each document for relevance—a demanding task that does not guarantee capturing all pertinent papers effectively. Future advancements in retrieval methods or literature screening software may help address these challenges.

## 5 Conclusion

This study highlights the rapid growth of research on RBPs in osteosarcoma, fueled by advances in epitranscriptomics and translational oncology. Future investigations should leverage international collaborations and emerging technologies to integrate mechanistic insights with clinical applications, ultimately improving patient outcomes.

## Data Availability

The raw data supporting the conclusions of this article will be made available by the authors, without undue reservation.
